# Determinants to implementing a new early literacy screener: Barriers and facilitators

**DOI:** 10.1007/s11881-025-00333-2

**Published:** 2025-07-16

**Authors:** Stephanie Tatel, Laura Jo Darcy, Emily J. Solari, Carlin Conner, Latisha Hayes, Latara Lampkin, Jamie DeCoster, Katie Wilburn, Cassidi Richmond

**Affiliations:** 1 Department of Curriculum, Instruction, and Special Education, University of Virginia, Charlottesville, VA USA; 2 Florida Center for Reading Research, Florida State University, Tallahassee, FL USA

**Keywords:** Determinants, Implementation, Language, Literacy, Reading, Screener

## Abstract

Early literacy screening is critical for identifying students at risk for reading difficulties and enabling timely intervention. However, the implementation of new screening tools often encounters significant barriers that hinder effective adoption. This mixed-methods study investigates the barriers and facilitators reported by educators during the early implementation of a new early literacy and language screener. Data from survey (*n*=694) and focus groups (*n*=39) were thematically coded using the Consolidated Framework for Implementation Research (CFIR) to identify determinants of successful adoption. Findings revealed four key themes influencing implementation: ease of administration, understanding of the screener, trust in the data, and use of data for instructional decisions. Crosscutting themes included the critical role of reading specialists and the challenges posed by simultaneous curriculum and assessment initiatives. Barriers such as time constraints, technological challenges, and insufficient understanding of language-based subtests were identified, while robust inner-setting supports and effective communication emerged as facilitators. These results underscore the importance of accessible and timely professional development, iterative tool refinement, and strategic leadership in scaling evidence-based practices. Future research should explore longitudinal impacts and strategies to integrate such innovations seamlessly into schools’ instructional ecosystems.

**Supplementary Information:**

The online version contains supplementary material available at 10.1007/s11881-025-00333-2.

Early literacy screening is a critical focus in education policy and practice due to its role in determining which students are at risk for reading difficulties (Catts & Hogan., 2020; Catts et al., 2015; Fletcher et al., 2020; Gaab & Petscher, 2022; Miles et al., 2018; O’Connor et al., 2014; Solari et al., 2021). Research demonstrates that early identification, through evidence-based screening, and intervention are essential, as reading difficulties often persist without adequate instruction (Francis et al., 1996; Juel, 1988). Research findings highlight the importance of effective implementation of psychometrically strong screeners (Komesidou et al., 2022; Morgan et al., 2024). This mixed-methods study utilizes an implementation science framework to investigate the reported determinants (i.e., barriers and facilitators) of implementation of use of an evidence-based screening system, of educators who participated in the early adoption phase of the Virginia Language & Literacy Screening System (VALLSS; Solari, Conner, & Soland, 2024).

## Importance of early literacy screening

Literacy screening allows for early identification of students at risk for reading difficulties, enabling timely interventions that can significantly improve reading outcomes (Catts et al., 2015; Connor et al., 2013; O’Connor et al., 2014; Lovett et al., 2017; Shaywitz et al., 2008). Extant research suggests that intensive, evidence-based reading interventions can reduce or eliminate reading difficulties when provided during a child’s first two years of school (Solari et al., 2021). Implementing screening as early as preschool can shift educational practices from a reactive to a proactive model, addressing potential literacy difficulties before they escalate (Torgesen et al., 1999). Additionally, early identification and support can mitigate the negative impacts of reading disabilities and difficulties on a student’s academic, social, and emotional development (Catts & Hogan, 2020; Denton et al., 2008).

Early literacy screening involves the use of empirically supported tools to assess students’ reading readiness and identify those at risk for reading difficulties as early as preschool (Torgesen et al., 1999; Catts et al., 2015). This process includes evaluating specific skills that predict reading success, such as phonological awareness, letter-sound knowledge, oral reading fluency, and oral language skills (Scarborough, 2001). By using reliable screening tools and implementing a structured screening protocol, educators can gather reliable data to inform targeted interventions and support for at-risk students (Denton et al., 2008).

Over the last four decades, literacy screening practices for reading difficulties have evolved, with research identifying various risk factors associated with phonological awareness, letter knowledge, and oral language (Catts et al., 2015), laying the groundwork for current screening methods by identifying key predictors of reading disabilities and introducing statistical decision theory to improve risk prediction (Connor et al., 2014). Today’s screeners utilize complex statistical analyses, including item response theory, to select the best items, optimizing prediction while reducing the burden on teachers for scoring assessments themselves (Fletcher et al., 2020).

As the importance of early identification and prevention in addressing reading difficulties gains recognition (Petscher et al., 2019), screening measures have become more widely available and easier to administer. Current literacy screeners facilitate a seamless connection between screening and instruction, empowering teachers to respond effectively to student needs (Barnes & Peltier, 2022). Furthermore, the integration of screening with diagnostic and progress monitoring tools supports ongoing analysis of student development, allowing teachers to adjust instruction based on individual progress (Fletcher et al., 2020).

## Legislated literacy change

Policy and accountability measures have driven increased state literacy policies in recent decades (Coburn, 2001), reaching into classrooms more directly than ever before, influencing curriculum, assessment, and teaching methods (McGill-Franzen, 2000). The current wave of reforms, often labeled as “Science of Reading” policies, continues to shape reading instruction infrastructure and implementation (Woulfin & Gabriel, 2022). Over the past six years, reading legislation broadened to encompass more than 40 states, including teacher preparation, professional development, assessment, family engagement, and student supports (Neuman et al., 2023). While current legislative language varies in the way the phrase “Science of Reading” is defined, most states describe the need for explicit, systematic, and rigorous instruction in reading. Many bills reference reading skills as defined by the five pillars outlined in the National Reading Panel Report (2000): phonemic awareness, phonics, fluency, vocabulary, and text comprehension. State mandates play a crucial role in the early screening for reading risk by requiring assessments in Kindergarten through Grade three (Fletcher et al., 2020; Schwartz, 2024). In fact, an emphasis on screening and assessment to monitor student performance is a central aspect of the legislation in almost all states. The Virginia Literacy Act (VLA) (SB 616), passed in 2022 and expanded in 2023, mandates the development and use of an approved literacy screener for K-8 students in Virginia. VALLSS, which began development before the VLA was passed, is the state-approved mandated literacy screener for all student in public schools in Virginia (Virginia Department of Education [VDOE], 2023).

Local enactment of these policies varies, with educators interpreting and implementing them based on their roles and contexts (Woulfin & Gabriel, 2022), further illustrating the complex interplay between policy and classroom practice in reading instruction. Much has been written about the change process in classrooms in response to legislated reading reform (Spillane, 1998; Coburn, 2006; Lightner et al., 2021). Teachers navigate complex communication channels, requiring nuanced judgment when interpreting messages from multiple sources to guide their instructional decisions (Spillane, 1998).

Messages from authoritative sources often carry more weight, shaping teachers’ understanding of required changes and influencing their enactment of literacy reforms (Coburn, 2006). Relational trust and a shared understanding of the reform vision among teachers, literacy coaches, and principals are also critical, and inconsistent messaging contributes to variance in policy enactment across schools and districts (Lightner et al., 2021; Woulfin, 2015; Woulfin & Gabriel, 2022). Spillane (1998) found that even within a single district, reading reform implementation varied when central office leadership lacked a unified vision for change. Middle leaders, such as reading specialists and literacy coaches, can bridge the gap between policy and practice, guiding teachers and supporting the sustainability and effectiveness of literacy reforms (Lightner et al., 2021).

## Determinants of implementation of the screener

Despite the evidence suggesting the value of a comprehensive literacy and language screener, educator use of the data is not guaranteed. While literacy screeners provide valuable insights for designing targeted instruction to enhance literacy outcomes (Solari et al., 2024), large-scale and sustained implementation of these tools with fidelity comes with challenges, including training, administration, and interpretation of results (Brown et al., 2021; Komesidou et al., 2022). As such, it is important to understand the determinants, or the facilitators of and barriers to, the implementation of the screener. This study’s surveys and focus groups examined implementation determinants to enhance screener adoption and advance implementation science.

Implementation science provides a framework to address uptake barriers by systematically analyzing contextual factors influencing adoption, including individual, organizational, and policy environments. To advance knowledge translation (Solari et al., 2020), implementation science investigates methods for consistently translating proven practices into widespread, sustainable use across multiple settings (Damschroder, 2020). Developing and applying targeted implementation strategies, such as professional development and feedback mechanisms, can overcome identified barriers.

## Theoretical framework

**Fig. 1:**
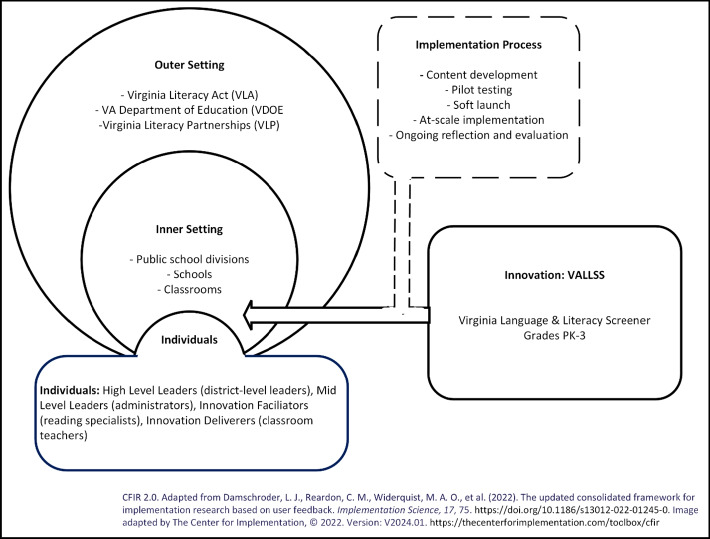
CFIR domains contextualized

Careful attention to the implementation process is necessary to support the translation of evidence-based practices into regular use in schools. The Consolidated Framework for Implementation Research (CFIR; Damschroder et al., 2022) describes barriers to and facilitators of implementation across four key domains: (a) the innovation, defined as this screener; (b) the outer-setting, which includes the VLA and the institutions supporting its implementation; (c) the inner-setting, comprised of school divisions and schools; and (d) individuals, including district-level leaders, school-level leaders, reading specialists, and teachers. Figure 1 illustrates the CFIR framework within this study’s implementation context. By applying this determinants framework, the screening developers can pinpoint key factors that impact implementation success and create solutions.

## Current study

This study surveyed screener users to capture feedback on the new screening system’s implementation. The survey explored how teachers use the system to collect and use student data, identify implementation challenges with the technology platform, and understand user experiences. The goal was to develop responsive resources and improve the screening system for future administrations.

The screener was developed in collaboration between the Virginia Department of Education (VDOE) and Virginia Literacy Partnerships (VLP), housed within the University of Virginia’s School of Education and Human Development. VALLSS includes subtests assessing decoding (word reading), linguistic (language) skills, and rapid naming (processing speed). VALLSS is unique because it is one of the only literacy screeners that includes language screening measures and is implemented at the state level. VALLSS serves a dual purpose: first, it identifies students at risk for reading difficulties, and second, it provides instructionally relevant information on foundational decoding and language skills.

This study aims to identify barriers and facilitators of screening system implementation across a representative sample of VA school divisions and describe educator uptake during the initial launch. This study asks the following research questions: (1) What barriers and facilitators to administering a new early literacy screener do educators report?; (2) What barriers and facilitators to using data to make instructional decisions do educators report?; and (3) How do these barriers and facilitators relate to the experience of administering the screening system and using the data for instructional purposes?

## Methodology

### Study context

To investigate educators’ and instructional leaders’ experiences, understanding, perceived value, and use of the screener, and to be responsive to any barriers experienced by individuals who were early adopters of VALLSS, data was collected from educators who participated in the initial launch of VALLSS. A subset of 17 school divisions agreed to participate in the early adoption of VALLSS during the 2023–2024 school year, and feedback from them was used to refine the screener. Survey and focus groups were conducted with teachers, specialists, and instructional leaders with representation from all eight geographic regions in Virginia.

### Participants

The survey was distributed via Qualtrics to 6119 personal email addresses. Teachers and instructional leaders who were early adopters of VALLSS during the 2023–2024 school year were invited to participate. After removing undelivered email invitations, a total of 6056 users were invited to complete the survey. Of this number, 827 participants consented and completed some items in the survey. The resulting response rate was 13.66%. The survey remained open for 20 days, and two email reminders were sent during that period.

Classroom teachers (74.4%) from Kindergarten through Grade 3 predominantly comprised the respondents. Almost all were female (96.3%) and white (87.8%), and a majority had a master’s degree. Almost all held a professional license, and most had 10 years or more of teaching experience. Quantitative analyses focused on a subset of 694 survey respondents, as seen in Table 1, who indicated that they assessed students and/or reviewed screening data, and excluded respondents indicating they supported these activities as an instructional leader. Respondents in the analytic sample are referred to as “educators,” since the sample includes classroom teachers, reading specialists, special educators, English as a second language (ESL) teachers, and reading intervention teachers.

**Table 1:** Educator roles of survey respondents and focus group participants

**Survey Respondents**	***n* (percent)**
Classroom educator	516 (74.4%)
ESL teacher	14 (2.0%)
Special educator	22 (3.2%)
Reading specialist	100 (14.4%)
Coach	24 (3.5%)
Other	18 (2.6%)
**Total**	**694 (100%)**
**Focus group participants**	***n* (percent)**
Classroom teacher	17 (43.5%)
ESL teacher	1 (2.6%)
Interventionist	7 (17.9%)
Reading specialist	5 (12.8%)
Instructional leader/coach	6 (15.4%)
School administrator	1 (2.6%)
Division coordinator	1 (2.6%)
Other	1 (2.6%)
**Total**	**39 (100%)**

Focus group participants were contacted via email from survey respondents who volunteered to participate. Ten focus groups were conducted including 31 educators and eight instructional leaders. Supplementary Materials Table 1 describes the roles per each focus group. Focus group participants were all female and almost entirely white and non-Hispanic.

### Data collection and measures

#### Survey

A team of reading researchers and VALLSS implementation coaches created the survey. Following Bandalos’ (2018) guidelines, the team iteratively developed items using precise language, avoiding negatives and lengthy sentences. The team drafted and refined items based on key CFIR framework constructs, carefully considering question types and quantity. The final survey was built with a branching structure so administrators and teachers responded to different items. The survey was distributed via email to every educator with an account in the screening system platform. Survey data described the study sample, and was analyzed through the CFIR framework and triangulated with focus group findings to reveal potential topics for deeper exploration among initial launch educators.

#### Focus groups

Focus groups further probed survey findings. Focus groups provide detail and depth into participants’ insights and experiences and how they make sense of those experiences (Hall, 2020). In focus group discussions, participants can reflect, discuss counterarguments, and change their opinions, revealing convergence and divergence within the group and generating different ideas unlikely to emerge through individual interviews (Hall, 2020; Lauri, 2019). Group moderators were trained to respond neutrally to participants and encourage diverse opinions by directly questioning participants. Participants were not shy about sharing different perspectives; in fact, they were interested in hearing about participants’ experiences from different school divisions. Researchers identified consensus in focus groups by noting common ideas, observing body language and Zoom reactions, and paraphrasing key points to confirm agreement and check for disagreement (Krueger & Casey, 2014).

The semi-structured focus group protocol was developed using the CFIR framework with a team of researchers and practitioners. Each focus group consisted of 3–7 participants, with a mean group size of 4.3, and was conducted by two researchers. Reading specialists and interventions, K-2 classroom teachers, and division-level instructional leaders comprised the focus groups. Focus groups lasted 60–90 min on Zoom and were recorded and transcribed verbatim. Post-interaction reflections were completed to inform ongoing data collection and analysis.

Coding and analysis followed a multi-stage, iterative approach. Initially, a priori domains from the CFIR framework were used to organize the data, deductively code 20% of interview transcripts, establish reliability, and refine the coding framework (Damschroder et al., 2022). To minimize bias, multiple coders analyzed all transcripts. A team of 10 coders trained on the coding process and the mixed-methods software, Dedoose, achieved a median inter-rater reliability score of .71 with a range of 0.65–1.00 (Cohen’s kappa). Teams met weekly to discuss emergent themes and refine the framework. All transcripts were independently coded by pairs and further analyzed collectively to identify themes. The team used Dedoose tools, including application charts, co-occurrence matrices, and code cloud to support analysis and identify patterns in the data (Braun & Clarke, 2006).

Team-based analysis fosters collaboration, creativity, and inclusivity, enabling more nuanced data interpretations than individual analysis (Brower et al., 2019; Weston et al., 2001; Yin, 2014). Teams used internal documents (e.g., coding schedules, logs, and analytic notes) to track and guide analysis while clarifying interpretations and building consensus. During coding and analysis, teams revisited data to refine connections, highlight shared meanings of participant experiences, identify illustrative quotes and excerpts, and draw preliminary and final conclusions. They examined countervailing evidence and outliers to address complexities and challenge simple explanations, aligning with culturally responsive practices (Miles, Huberman, & Saldaña, 2014). Finally, preliminary findings were shared with key informants for member-checking (Eisenhart & Howe, 1992).

## Findings

Triangulation of survey and focus group data revealed barriers and facilitators associated with the innovation design, outer-setting, inner-setting, and individuals as conceptualized in the CFIR framework.

### Survey analyses

As seen in Table 2, survey respondents on average felt less prepared than prepared to assess students with VALLSS (with the mean falling between “unprepared” and “prepared”) and that VALLSS was somewhat difficult to implement (with the mean falling between “difficult” and “easy”). Major obstacles (time to test and managing other students) and minor obstacles (technology) presented barriers during VALLSS administration. Participants reported limited use of supports offered by the outer-setting (VLP), but those who used the supports rated them as “fairly helpful.”

**Table 2:** Experiences and determinants related to implementing VALLSS

Measure	Range	Mean (SD)	Median
Experience implementing VALLSS			
Feeling prepared to implement VALLSS	1 (very unprepared) to 4 (very prepared)	2.84 (.65)	3.00
Ease of implementing VALLSS	1 (very difficult) to 4 (very easy)	2.49 (.75)	3.00
Facilitators/barriers			
Number of VALLSS supports used	0 to 5	.97 (.94)	
Helpfulness of VALLSS resources	1 = Very unhelpful to 4 = Very helpful	3.11 (.49)	1.00
Minutes spent implementing VALLSS	1 = Under 30	2.63 (.85)	3.00
	2 = 30–45		
	3 = 45–60		
	4 = More than 60		
Days spent implementing VALLSS	1 = 1–5	2.63 (.98)	3.00
	2 = 6–10		
	3 = 11–15		
	4 = More than 15		
Rating of technical obstacles to implementing VALLSS (technology)	1 = Not at all an obstacle to 4 = Was a significant obstacle	1.95 (.66)	1.83
Rating of other obstacles to implementing VALLSS (time, managing other students and the testing situation)	1 = Not at all an obstacle to 4 = Was a significant obstacle	2.50 (.66)	2.57

Educators used both code-based and language-based data, with greater reliance on code-based data. As seen in Table 3, respondents reported feeling less prepared than prepared to use VALLSS data, viewed multiple VALLSS reports, but made limited use of VLP data-use resources. Most respondents reported they had support from their school/division, such as regular meetings with colleagues to review data.

**Table 3:** Experience and determinants related to VALLSS data-use

Measure	Range	Mean (SD)	Median
Experience implementing VALLSS			
Number of ways code-based data were used	0 to 9	3.49 (2.49)	3.00
Number of ways language-based data were used	0 to 9	2.82 (2.55)	2.00
Feeling prepared to use VALLSS data to inform teaching	1 (very unprepared) to 4 (very prepared)	2.62 (.76)	3.00
Facilitators/barriers			
Number of VALLSS reports viewed	0 to 8	3.83 (1.77)	4.00
Number of VALLSS data resources used	0 to 4	1.18 (1.20)	1.00
Number of data supports provided by school/division	0 to 4	1.66 (1.14)	1.00
How often participants met to review VALLSS data	1 = Weekly or almost weekly	2.23 (1.00)	2.00
	2 = Once or twice a month		
	3 = Less than once a month		
	4 = Once or twice a year		
	5 = Rarely		

Researchers examined the Spearman rank correlations of the VALLSS implementation experience measures with the measures of implementation facilitators and barriers. Spearman rank correlations were chosen because they are designed to work with ordinal scales and describe the presence, magnitude, and direction of the relations. As shown in Table 4, feelings of preparedness and implementation difficulty were unrelated to implementation time and weakly related to the number of supports used. Feelings of preparedness were strongly related to resource-helpfulness and the presence of both technical and other obstacles. As expected, reported feelings of preparedness and ease of use of the VALLSS data were positively related to the number of supports used and helpfulness of the resources and negatively related to the presence of technical or other obstacles.

**Table 4:** Spearman rank correlations of VALLSS implementation experience with implementation facilitators and barriers

	*ρ* (*ρ*2)	
	Preparedness for implementation	Ease of use
VALLSS supports used	.17** (.03)	.13** (.02)
Helpfulness of VALLSS resources	.31** (.10)	.32** (.10)
Minutes spent implementing VALLSS	−.14** (.02)	−.27** (.07)
Days spent implementing VALLSS	−.10* (.01)	−.22** (.05)
Rating of technical obstacles to implementing VALLSS	−.29** (.08)	−.36** (.13)
Rating of other obstacles to implementing VALLSS	−.31** (.10)	−.42** (.18)

*p < .05, **p < .005

Code-based data-use, language-based data-use, and feelings of preparedness for data-use were all positively related to the number of reports viewed, the number of data resources used, and the number of data supports provided by participants’ schools or divisions (see Table 5). Additionally, participants who met with others more often to review VALLSS data were more likely to use both code-based and language-based data to inform their instruction.

**Table 5:** Spearman rank correlations of VALLSS data-use with data-use facilitators and barriers

	*ρ* (*ρ*2)		
	Code-based data-use	Language-based data-use	Preparedness for data-use
Number of VALLSS reports viewed	.26** (.07)	.20** (.04)	.17** (.03)
Number of VALLSS data resources used	.23** (.05)	.18** (.03)	.11* (.01)
Number of data supports provided by school/division	.29** (.08)	.23** (.05)	.18** (.03)
How often participants met to review VALLSS data	−.14** (.02)	−.16** (.03)	−.04 (.002)

*p < .05, **p < .005

### Mixed-methods analyses

Researchers identified themes influencing teachers’ use of VALLSS screener data for instructional decisions, as described in Table 6. Findings suggest that educators’ assessment uptake for instructional decisions is influenced by their administration experience, comprehension of the assessment’s purpose and design, and their trust in the assessment’s data value. Threaded throughout these themes was evidence that reading specialists were key implementation facilitators.

**Table 6:** Themes and definitions

**Analytic theme**	**Definition**
Administering the screener	Educators can administer the screener correctly, efficiently and with minimal obstacles.
Understanding the screener	Educators understand the content measured by the screener and know how to interpret the data.
Trusting the data	Educators believe that the data is an accurate and valid reflection of their students’ strengths and needs.
Using the data	Educators easily use the data to make student-level decisions about instruction and intervention.

Implementing new practices across settings involves complex challenges (Waltz et al., 2019). These themes illuminate the complex transition process for teachers and schools implementing a new screener, while also identifying the critical contextual factors that influence both the screening tool’s implementation and subsequent data utilization.

#### Theme 1: Administering VALLSS

*Administering* is the extent to which educators administered the assessment accurately with minimal obstacles and adequate support. Educators reported that practical obstacles during testing (e.g., time constraints, student management, technology issues, task content) were mitigated by inner-setting resources, which varied widely.

### Time and managing other students

As shown in Table 2, survey respondents reported practical (“other”) obstacles to administering VALLSS, including time to test and managing other students. The extent to which teachers reported these obstacles was negatively correlated with their reports of ease of use, as shown in Table 4. Focus group participants corroborated that time and managing other students during testing were significant barriers, describing the testing process as “long” and “arduous.” Educators discussed the relationship between these two obstacles in terms of students’ limited ability to work independently during test sessions. Common examples from focus groups included expressions of frustration: “We are very stressed about being away from our kids” and “It was very, very difficult, and it was frustrating to just sit there in class and be like, ‘I need you to be quiet.’” Educators discussed testing time in terms of lost instructional time: “...every minute that we’re spending on assessment is taking away from instructional minutes. And so, really being very careful and figuring out... what is it that’s worth that sacrifice. But those instructional minutes are also valuable.”

Participants across focus groups commented on the data’s value, even as they questioned the sacrifice of instructional time. One example of this sentiment was stated, “There is great information to glean from the data that we are getting. But then, also you have to balance that out with the loss of instructional time.” Another participant shared, “You want all that useful data...but it did take so much time away from instruction.” One participant, however, noted that it was time well-spent for the data provided and stated, “It’s very justifiable to give it the time because of the amount of information you get from VALLSS.”

### Technology

Survey respondents reported technology barriers that influenced their perceptions of the ease of administering VALLSS (see Table 4). Focus group participants consistently echoed these challenges: “Visually, the website is very hard. Everything’s really small...Teachers had a really hard time with that.” Many focus group participants pointed out challenges with the overall layout such as font size and the lack of gridlines to visually connect the student name with the score.

### Perceived difficulty of VALLSS content

Common across the focus groups, participants described their perceptions of the screener content that affected the testing experience as sometimes “discouraging for the kids and for the teachers” and “extremely difficult.” To address this concern, focus group participants suggested discontinuation rules within and across subtests, and greater alignment with grade-level content (e.g., specific phonics features from a grade-level progression).

### Inner-setting support

Focus group participants overwhelmingly agreed that division- and school-level support was crucial for completing the screener. VLP emphasized that classroom teachers should personally administer the screener to their own students. One participant shared: “Our county really took to heart that the teachers should do everything. And so, when the teachers got stressed, we were not allowed to step in and give them a hand with any of it.” Commonly, participants reported that receiving coverage for classes from a substitute teacher or paraprofessional to administer VALLSS was helpful. Principals were frequently mentioned as providing this valued inner-setting support. One participant stated that, “Our principal worked really hard to give teachers some time [with coverage to test].” In addition to personnel support, there was consensus from focus groups that alternative testing spaces facilitated VALLSS administration to provide “a quiet space so that the student can concentrate on the assessment.” Most participants across the focus groups agreed that their school divisions expected teachers to complete the screener themselves, though at least two noted that some schools delegated testing to other staff. One instructional leader explained the rationale for shifting the responsibility for testing students from teachers, stating, “The testing itself takes such a long time...they’re missing a lot of instruction.”

### Inner-setting relational connections

Focus group participants repeatedly said that the support of peers and reading specialists facilitated VALLSS administration. One participant described how colleagues collaboratively troubleshot difficulties during assessment. “I feel like our teams and...our reading specialists, for us were just a huge source of support.” Another explained, “We did a lot of collaboration at our school...odds are somebody else had experienced a similar problem already and may already have the solution.” Focus group consensus overwhelmingly emphasized the importance of relationships, networks, and teams in supporting screener administration.

#### Theme 2: Understanding VALLSS

*Understanding* is defined as access to knowledge and information about administering the screener and interpreting the data.

### Knowledge and information from the outer-setting

Outer-setting resources from VLP supported understanding through a phone/email hotline and webinars. Many focus group participants noted positive interactions with VLP in hotline and office hours, describing staff as “very responsive,” “very patient,” and “very quick to solve all the problems.” Another educator said, “My team here was able to learn a lot from the office hours, and from the newsletters and emails we got from VLP...we got to ask a lot of questions...” Educators reported that VLP was interested in stakeholder feedback. One stated that VLP was “very open to suggestions that we had...so it was a very positive experience.”

### Inner-setting communication

Understanding was further facilitated by inner-setting communication that transmitted, translated, and disseminated information from the outer-setting. According to focus group participants, the most effective communication included all stakeholders, was delivered in a timely and targeted manner, and featured multiple information access points. One focus group participant noted, “We’re thoughtful on how we share information so that it doesn’t get lost in the shuffle, so we [created] a running document ...so whenever we received emails from VLP related to the assessment, we made sure to add those pieces of information to that document.” Weak communication negatively impacted understanding. One participant explained, “One of our facilitators in the district would find out, and then it would have to trickle down to us. So, you know...We were left to figure things out.” Focus group participants repeatedly endorsed reading specialists as central to facilitating communication and teachers’ understanding. One focus group participant explained that “in our district, you know, a lot fell on the reading specialists on how to teach teachers how to read the data.”

### Gaps in understanding

Some participants attributed a gap in understanding to a negative experience completing the VALLSS training modules. Some participants reported having sufficient time during pre-week hours to complete the training with support from a reading specialist, while others reported having to complete it on their own time, along with a list of other required online trainings for the beginning of the school year. Some participants also noted that their schools were implementing several new initiatives at once, further complicating completion of the online training for VALLSS.

Some focus group participants reported that it was easy to understand the content of the screener because of prior training in reading science. However, gaps in understanding were often linked to the language-based subtests. Several participants described scoring one subtest as “tricky” and “subjective.” Reading specialists reportedly addressed these issues by seeking clarity from VLP and sharing information with teachers. Participants also positively noted the guidance resources added to the VALLSS platform since the time of the fall testing window.

Educators often struggled to grasp the purpose of specific subtests, particularly those absent from the previous state screener (e.g., rapid automatized naming and language subtests). One participant noted, “we need a little more information about what the purpose of some of the subtests are.” Another observed that teachers with limited knowledge of reading science might not appreciate “how important all these different subtests are, and why they’re even there.”

A common theme was a desire for greater clarity about how risk indicators were calculated in the testing platform, especially when students had different levels of risk but seemed to perform similarly overall when examining raw subtest scores. For example, one educator expressed confusion about scoring, stating, “If we had more of a solid idea of the scores, and what has more weight, and understanding what exactly they mean, I think that we would be able to really truly understand it.” Another added, “I did all this work... But I don’t know how you put this all together...why this kid came out as moderate risk and this kid came out as low risk when their scores are pretty similar. It wasn’t obvious to me.”

Educators characterized this as a confusing barrier to setting instructional targets and responding effectively. They emphasized the need for clarity, with one suggesting, “Some understandings and something written, so that teachers can see what is an acceptable amount of growth. Like, where should a first grader be going towards midyear?” Participants overwhelmingly reported that interpreting and acting on the language data was a challenge; “If a kid doesn’t have high vocabulary fluency, other than like your daily exposure to words and text and interactions in the classroom... how do you help students in that specific area?” These challenges also affected teachers’ ability to communicate VALLSS results to families. As one noted, “Definitely, at the beginning of the year I really didn’t know how to communicate that to parents.”

#### Theme 3: Trusting VALLSS

*Trusting* is the degree to which educators believe the data are accurate and valid.

### Testing environment

Focus group participants acknowledged the challenges of administering VALLSS in the classroom where practical obstacles might affect students’ performance and impact scores. One participant noted that when the classroom teachers administer the passage retell task (in which the teacher reads a story aloud to the student) that “...by the twentieth student, they’ve heard it nineteen times...so that data [will] be skewed.” Another elaborated about this previous exposure saying, “We didn’t feel like it was accurate of our kids’ comprehension ability.” Educators noted testing locations free of distractions facilitated administration and, in turn, increased trust in the data. As one educator explained, “...asking them to do these types of tasks when there’s a million other things happening in the classroom was not gonna yield reliable data, so having that quiet space was helpful.”

### Perceptions about VALLSS content

Every focus group voiced concern with specific aspects of VALLSS content. Some participants noted the length and background knowledge demands for the listening comprehension passages. These perceived problems influenced their belief in the value of the data. One educator shared, “I did nothing with the retelling data, because I thought the story was extremely too long... my retelling looks much worse now than it did at the beginning, and I know they’ve gotten better with retelling.”

Educators also questioned the data when they perceived that item content did not match their instructional progression or exceeded their expectations for grade-level proficiency. One teacher explained, “It looks like their scores are low...but it’s stuff that we haven’t taught.” Another educator reported, “On the encoding, it’s not assessing first grade skills... so even that data doesn’t really help me.” These reports indicate a need for further professional development on the purpose of a screener versus a curriculum-based formative assessment.

### Unexpected risk determinations

In general, educators noted surprise at the number of students identified as high risk. One stated, “So many kids were identified this year with VALLSS that we were not expecting.” Educators consistently expressed a desire for greater transparency regarding how risk indicators were calculated in order for them to more fully trust the data. One participant articulated this intersection of understanding and trust, “I’m not sure how to look at the [Band of Risk], because I don’t know the criteria, so I don’t necessarily know how to process it all, because it’s hard to figure out. Why is this kid a high kid, and this kid is a moderate kid?” Another questioned, “What are we using to find this mysterious band of risk? Nobody could ever explain that to us.” Teachers were familiar with using the risk indicator for intervention assignment but wanted to understand its calculation. The Bands of Risk, calculated using Item Response Theory (IRT), could not be manually computed by teachers, unlike the previous screener which used benchmarks calculated by scores based on raw subtest summaries.

#### Theme 4: Using VALLSS data

*Using* the data concerns how easily and readily educators use the data for instructional decisions.

### Compliance with early intervention mandates

State requirement drove the use of screener data, most commonly to identify students scoring in the high-risk band for state-mandated intervention. One educator explained using the risk bands “to determine which students will be seen by the EIRI [Early Intervention Reading Initiative] tutors.” This sentiment was echoed by another: “We used the high-risk band as a conversation starter and accountability piece with those schools… you do need to do an intervention, you do need to do a plan, and yes, we’re going to check!’” The risk bands for mandated services are based on code-based subtests, which appeared to promote greater use of code-based over language-based data. One educator commented, “… knowing that they’re being flagged for the code[-based] tasks, I did not use the language comprehension scores or asterisks [instructional indicators] nearly as much.” In contrast to the fall initial launch window, the midyear assessment was shorter but did not include risk bands. Educators reported that while they appreciated the reduced length, they missed having the guidance provided by the risk bands. Using data for instructional planning

In general, survey respondents reported feeling prepared to use VALLSS data for instructional decisions. One participant noted that the instructional indicators “showed me which areas to focus on with that small group follow up.” However, gaps in educators’ understanding of VALLSS stymied the use of its data to inform instruction. One teacher said very clearly that “knowing the purpose would help us use the data.” Another participant explained, “We didn’t have, kind of, a lens through which to look at the data, or at least we didn’t have a lens that we felt very comfortable with and that we could easily kind of adjust our thinking to.” Many participants reported they used additional code-based assessments, either by choice or by mandate, because they did not fully understand the new screener and were “...looking at getting a better feel for the data this year.”

Understanding how to use VALLSS data in place of outdated “reading level” data was a barrier for some educators. One stated that they could not use the data to plan small group instruction because “we didn’t get a reading level from the kids.” Lacking familiar milestones, educators had difficulty communicating students’ proficiency to families. “Our parents are used to having a level, and while we’re trying to, you know get away from a D or an F or whatever [reading level], they still want to know if they’re on grade level, or are they above or below grade level…?”

### Reading specialists leading the shift

As shown in Table 5, survey data suggested that inner-setting systems, such as regular data meetings with colleagues, facilitated teacher uptake of VALLSS data for decision-making. Focus group participants consistently recognized that reading specialists were key facilitators for VALLSS data-use. One teacher explained, “We meet individually with our reading specialist to go over all the data... to analyze all the different pieces of data and make instructional decisions ...”

Reading specialists participating in the focus groups often described their leadership for VALLSS data-use. One focus group participant noted, “The instructional indicators were huge for us when it came to.... focusing on targeted small group and .... making that shift in at my school. It led to a lot of natural coaching cycles for me and at my school to help teachers shift to targeted instruction…” Another explained, “Teachers felt like their instruction, as we’re switching to science of reading, really matched the screener...we saw growth and our teachers were pleasantly surprised and happy to see that.”

Schools with strong inner-setting systems, supported by effective reading specialists, reported that VALLSS supported data-based decision-making. One participant explained, “We really were intentional with setting up data meetings to really dive in and look at the data, and then using a lot of what the VLP course had given the reading specialists....on how to interpret that data and how to use that to target your instruction.”

## Discussion

The current study sought to understand and respond to educators’ reports of determinants to administering a new literacy and language screener and using the data for instructional decisions. The new screening system is required by comprehensive literacy legislation passed in 2022 and expanded in 2023. Longitudinal studies show that early reading difficulties are likely to persist, leading to later reading difficulties when students do not receive adequate reading instruction (Francis, et al., 1996; Juel, 1988). While the justification for screening is well established, implementing it at scale is highly complex and may encounter barriers related to preparation, administration, and data-use (Bauer & Kirchner, 2020; Komesidou et al., 2022). Investigating the processes and factors supporting the implementation and maintenance of early screening for reading risk may inform effective implementation efforts in the future (Petscher et al., 2020). This study offers a unique perspective on the initial implementation of a state-wide literacy and language screening system.

In this mixed-methods study, four themes emerged about barriers to and facilitators of implementing the new screener: (1) Administration of the screening system with ease, (2) Understanding the screening system’s content, (3) Trusting the data produced by the screening system, and (4) Using the data for student-level instructional decisions. Threaded throughout these themes was evidence that reading specialists acted as key implementation facilitators. Additionally, and consistent with the research on organizational support for changing practices in schools (Aldridge & McLure, 2024) uptake of new practices is highly dependent upon the level of preparation and support in each school, and the number of new practices being implemented at once.

### Reported barriers to implementing and using a language and literacy screener

Time is a frequently cited barrier during implementation of new innovations (Barrett-Tatum & Ashworth, 2021) and can be particularly significant where teachers feel they lack the time to learn about, adapt to, or integrate new evidence-based interventions into their routines (Bauer & Kirchner, 2020). Educators reported that the screener’s length (i.e., the time to administer the screener) was a significant barrier. Based on analyses of VALLSS pilot and initial launch item-performance, modifications were made that reduced administration time, including making some subtests optional, and incorporating discontinuation rules for several subtests. Supportive, user-friendly, and integrated technology is essential for implementing evidence-based practices (Damschroder et al., 2022). To address this element of the innovation, VALLSS developers engage in continuous cycles of development, refinement, and enhancement informed by both internal processes and user feedback. Technology was a commonly reported barrier during the initial launch, with educators facing difficulties using the assessment platform and accessing reports. Since that time, user feedback drove improvements to the assessment platform include a redesigned user interface, clearer navigation, and enhanced report formatting.

Complex innovations that require changes in practice face uptake barriers (Bauer & Kirchner, 2020). Educators expressed concerns about the complexity and content of screener tasks, citing issues such as the number of tasks, lengthy subtest instructions, complex scoring procedures, and perceived flaws in phonics features and listening comprehension topics. These concerns affected their perceptions of the screener’s validity and trustworthiness. In addition, while educators were familiar with code-based assessments, they lacked experience with language comprehension assessments, posing challenges for adoption. The development of robust training supports that are now widely accessed by educators was underway during this initial launch. These supports include a state-wide Canvas course, tutorials for each subtest, video demonstrations of teachers administering the screener to students, and a collection of resources on the testing platform including one-pagers on communicating with families, scoring tips, and data-interpretation.

Finally, educators reported that the new screener was one of many changes they had to make in preparation for the VLA to take effect. The school divisions involved in the initial launch of VALLSS represented various stages of reform enactment required by the new legislation. Some educators felt overwhelmed by simultaneously implementing new curricula, instructional strategies, professional development, and a new literacy screener. Too many changes all at once can itself present a barrier to the implementation of any one initiative (Aldridge & McLure, 2024).

### Reported facilitators to implementing and using a language and literacy screener

Educators consistently credited inner-setting structures and supports as important to the successful administration of the screener and data-use. Research also shows inner-setting supports moderate implementation by providing the structural, relational, and cultural foundations necessary for effective change, and by leveraging external motivations and partnerships for successful innovation adoption (Damschroder et al., 2022). Participants who described strong inner-setting supports for the implementation of the screener frequently attributed the success of those supports to their principal and to division literacy leaders. The reading specialist is also a crucial role to provide effective inner-setting supports to teachers. In fact, this was by design of the VLA, which includes a requirement of adequate reading specialist staffing to coordinate and oversee interventions for students identified by VALLSS, and to support teachers and families. Educators frequently reported that the reading specialists at their school were key to communication, access to knowledge and information, and direct support for administering the screener and data-use. The reading specialists leveraged positive relationships within the school that many educators reported as a powerful facilitator to implementation. Participants consistently characterized reading specialists as facilitating effective implementation of the screener and helping to bridge the gap between policy and practices (Lightner et al., 2021).

Educators who described a robust multi-tiered system of supports in their school also credited the VALLSS data with identifying students who most need intervention, and guiding design of interventions. In these cases, VALLSS was compatible with the schools’ structures and processes. The existing culture within schools and divisions, including their openness to change and readiness to adopt new practices, positively impacted these implementation efforts (Bauer & Kirchner, 2020).

Educators reported that outer-setting technical support provided by VLP (e.g., email and phone hotline) was critical. In some cases, teachers accessed the hotline directly, while in other cases the reading specialist called the hotline on behalf of teachers. The hotline gave educators a tool for immediate problem-solving. This real-time support is crucial for maintaining momentum and ensuring successful implementation (Damschroder et al., 2022). VLP also held weekly virtual office hours for teachers, providing yet another connection between inner- and outer-settings, a practice known to be associated with increased professional knowledge and implementation success (Damschroder et al., 2022).

### Implications

The findings from the current study underscore the importance of leveraging implementation science frameworks to prepare for and improve implementation of a new screening system at scale. The use of appropriate and standardized early screening can aid in the early detection of learning difficulties, allowing for timely interventions that improve children’s learning opportunities, which is essential for addressing potential developmental delays before they affect a student’s educational path (Miles et al., 2018). However, the existence of an effective screener does not guarantee its correct and consistent use. Implementation science provided a determinant framework to address uptake barriers by systematically analyzing contextual factors influencing adoption. Iteratively developing and applying targeted implementation strategies, such as professional development and feedback mechanisms, can overcome identified barriers and facilitate and sustain use of the screener. Ongoing professional development is required to support the administration, understanding, trust, and use of the screener data. Findings from this study can broadly inform other implementation efforts of evidence-based literacy and language practices, which is especially important as states across the country are enacting legislation to improve instruction, curriculum, assessment and professional development in literacy.

To encourage adoption of evidence-based literacy practices, research teams should communicate clearly and simply with collaborators about the scope of the screener and the research process (e.g., screener development typically starts with a larger pool of items and then uses data from field testing to eliminate items). During implementation, gathering feedback from users guides innovative modifications. In this study, the research team’s use of implementation science provided feedback from users that informed changes to the administration of the screener and the technology. It is noteworthy that focus group participants consistently expressed enthusiasm and gratitude to be included in the process.

This study supports the identification of a key implementation facilitator, or middle leader (Lightner et al., 2021), such as the reading specialist. Future research using the CFIR framework should examine how this individual’s success relies on strong school leadership. Participants frequently noted that communication about VALLSS primarily came through the reading specialists, with few people noting the role of principals in communication. Because the reading specialist is central to the VLA, information from the outer-setting to the inner-setting flowed primarily through this individual in the initial launch. Since then, training and communication have been provided to principals. School administrators have an important role to play in coherent implementation. They are positioned to message reforms to their staff in a way that integrates initiatives and sustains change (Fullan & Quinn, 2015). Furthermore, the reading specialist lacks positional power, thus limiting their influence among their peers. The principal and reading specialist can collaborate to provide support, drive progress, and clarify the specialist’s role. These findings underscore the importance of planful implementation efforts to translate evidence-based assessment into practice.

### Limitations

The findings of the current study should be discussed within the context of its limitations. First, our survey and focus group sample lacked racial and ethnic diversity making it not fully representative of the state K-3 public school teacher population. Second, the study relies on the self-reports of educators who voluntarily participated. The use of focus groups can also potentially impede the disclosure of information if the questions are perceived as personally or professionally sensitive or would otherwise be divulged in individual interviews.

## Conclusion

This study provides insights into the barriers and facilitators to successful implementation of VALLSS and provided VLP with information to improve educator experience when administering and interpreting VALLSS. Educators identified challenges related to time, technology, and understanding the screener’s complexity, which influenced their perceptions of its validity and usability. However, robust inner-setting supports, particularly the role of reading specialists, emerged as key facilitators in overcoming these obstacles. The results highlight the necessity of clear communication, professional development, and iterative improvements to resources related to using the screener data, the new platform through which the screener is accessed, and screener implementation processes to ensure effective data-use. Furthermore, the findings underscore the importance of considering the context of schools and classrooms when planning for implementation of new assessments. This research contributes to the growing field of implementation science and supports the use of evidence-based tools to improve early literacy outcomes for all students.

## Supplementary Information

Below is the link to the electronic supplementary material.

Supplementary file1 (DOCX 18 KB)

## Declarations

This paper was prepared using data provided under a contract with the Virginia Department of Education. The content does not necessarily reflect the views or policies of the Virginia Department of Education, the Virginia Board of Education, or the Commonwealth of Virginia. Consequently, the Virginia Department of Education, the Virginia Board of Education, and the Commonwealth of Virginia are not responsible for the paper’s content or any loss suffered due to the use of such content. Moreover, the mention of any trade names, commercial products, or organizations in this paper is not an endorsement of any of these entities by the Virginia Department of Education, the Virginia Board of Education, or the Commonwealth of Virginia.
