# Supplementary material for: Determinants to implementing a new early literacy screener: Barriers and facilitators
Source: Ann Dyslexia. 2025 Jul 16;75(3):524–46. doi: 10.1007/s11881-025-00333-2 (PMC12662938; doi:10.1007/s11881-025-00333-2)
Supplement: Supplementary file 1 — Supplementary file1 (DOCX 18 KB) [file 11881_2025_333_MOESM1_ESM.docx]

**Supplemental Online Materials *VALLSS Additional Information***

https://literacy.virginia.edu/

**Supplemental Online Materials Table S1. *Focus Group Participant Roles***

| **Focus Group** | ***n*** | **Participant Grade and Roles** |
| --- | --- | --- |
| FG1 | 4 | Grade 1 Teacher (n=3), Grade K Teacher (n=1) |
| FG2 | 5 | Reading Specialist (n=1), Grade 1 Teacher (n=2), Grade K Teacher (n=1), District Reading coach (n=1) |
| FG3 | 7 | Grade K Teacher (n=2), Reading Specialist (n=2), Teacher (n=1), District literacy coach (n=1), Grade 2 Teacher (n=1) |
| FG4 | 6 | Grade K Teacher (n=2), Grade 1 Teacher (n=10, Grade 2 Teacher (n=1), Teacher (n=1), Reading Specialist (n=1) |
| FG5 | 4 | Grade K Teacher (n=1), Grade 2 Teacher (n=2), Teacher (n=1), ESL Teacher (n=1) |
| FG6 | 3 | Grade K Teacher (n=1), Teacher (n=2) |
| FG7 | 3 | Reading Specialist (n=2), District Reading Specialist (n=1) |
| FG8 | 3 | Reading Specialist (n=1), District Special Education Supervisor (n=1), District Literacy Coach (n=1) |
| FG9 | 4 | Principal (n=1), School Psychologist (n=1), District Literacy Coach (n=1), District Intervention Specialist (n=1) |
| FG10 | 3 | Reading Specialist (n=3) |

*M* = 4.2; Range 3-7

**Supplemental Online Materials Table S2. *Survey participants other demographics***

| Grade | **n (Percent)** |
| --- | --- |
| Kindergarten | 163 (23.5%) |
| 1^st^ | 152 (21.9%) |
| 2^nd^ | 149 (21.5%) |
| 3^rd^ | 52 (7.5%) |
| Unreported | 178 (25.6%) |
| Education | **n (Percent)** |
| High school diploma or GED | 1 (0.1%) |
| Associate’s degree | 3 (0.4%) |
| Bachelor’s degree | 264 (38.0%) |
| Master’s degree | 393 (56.6%) |
| Education Specialist degree | 26 (3.7%) |
| Doctoral degree | 5 (0.7%) |
| Unreported | 2 (0.3%) |
| License | **n (Percent)** |
| Provisional | 38 (5.5%) |
| Collegiate | 263 (37.9%) |
| Postgraduate | 356 (51.3%) |
| None of the above | 21 (3.0%) |
| Unreported | 16 (2.3%) |
| Years of experience | **n (Percent)** |
| 1 | 24 (3.5%) |
| 2-5 | 129 (18.6%) |
| 6-10 | 127 (18.3%) |
| More than 10 | 411 (59.2%) |
| Unreported | 3 (0.4%) |
| Gender | **n (Percent)** |
| Female | 688 (96.3%) |
| Male | 9 (1.3%) |
| Unreported | 17 (2.4%) |
| Race/ethnicity | **n (Percent)** |
| Black or African American | 22 (3.2%) |
| Hispanic | 16 (2.3%) |
| White | 609 (87.8%) |
| Multiracial or other | 15 (2.2%) |
| Unreported | 32 (4.6%) |
